# Sympathetic Ophthalmia after Vitreoretinal Surgery without Antecedent History of Trauma: A Systematic Review and Meta-Analysis

**DOI:** 10.3390/jcm12062316

**Published:** 2023-03-16

**Authors:** Matteo Ripa, Georgios D. Panos, Robert Rejdak, Theodoros Empeslidis, Mario Damiano Toro, Ciro Costagliola, Andrea Ferrara, Stratos Gotzaridis, Rino Frisina, Lorenzo Motta

**Affiliations:** 1 Department of Ophthalmology, William Harvey Hospital, East Kent Hospitals University NHS Foundation Trust, Ashford TN24 0LZ, UK; matteof12@gmail.com (M.R.);; 2 Department of Ophthalmology, Queen’s Medical Centre Campus, Nottingham University Hospitals, Nottingham NG7 2UH, UK; 3 Department of General and Pediatric Ophthalmology, Medical University of Lublin, Ul. Chmielna 1, 20079 Lublin, Poland; 4 Department of Ophthalmology, Stoneygate Eye Hospital, Leicester LE2 2PN, UK; 5 Eye Clinic, Public Health Department, University of Naples Federico II, 80133 Naples, Italy; 6 Eye Clinic, Department of Neurosciences, Reproductive and Dentistry Sciences, University of Naples Federico II, 80131 Naples, Italy; 7 Department of Ophthalmology and Neuroscience, Medical School, University of Bari “Aldo Moro”, 70121 Bari, Italy; 8 My Retina Athens Eye Center, 11528 Athens, Greece; 9 Ophthalmology Unit of Surgery, Department of Guglielmo da Saliceto Hospital, 29121 Piacenza, Italy

**Keywords:** pars plana vitrectomy, panuveitis, sympathetic ophthalmia, vitreoretinal surgery

## Abstract

Background: To evaluate the morbidity frequency measures in terms of the cumulative incidence of sympathetic ophthalmia (SO) triggered by single or multiple vitreoretinal (VR) surgery procedures in eyes without an antecedent history of trauma and previous ocular surgery, except for previous or concomitant uneventful lens extraction, and to further investigate the relationship between VR surgery and SO. Methods: A literature search was conducted using PubMed, Embase, and Scopus from inception until 11 November 2022. The Joanna Briggs Institute (JBI) critical appraisal checklist for the case series and the Newcastle–Ottawa Scale were used to assess the risk of bias. The research was registered with the PROSPERO database (identifier, CRD42023397792). Meta-analyses were conducted using the measurement of risk and a 95% confidence interval (CI) for each study. Results: A random-effect meta-analysis demonstrated that the pooled cumulative incidence of SO triggered by single or multiple VR surgery procedures in eyes without an antecedent history of trauma and previous ocular surgery, except for previous or concomitant uneventful lens extraction among patients who developed SO regardless of the main trigger, was equal to 0.14 with a CI between 0.08 and 0.21 (I^2^ = 78.25, z: 7.24, *p* < 0.01). The pooled cumulative incidence of SO triggered by single or multiple VR surgery procedures in eyes without an antecedent history of trauma and previous ocular surgery, except for previous or concomitant uneventful lens extraction among patients who underwent VR surgery, was equal to 0.03 for every 100 people, with a confidence interval (CI) between 0.02% and 0.004% (I^2^ = 27.77, z: 9.11, *p* = 0.25). Conclusions: Despite postsurgical SO being a rare entity, it is a sight-threatening disease. VR surgery should be viewed as a possible inciting event for SO and considered when counseling patients undergoing VR surgery.

## 1. Introduction

Sympathetic ophthalmia (SO) is a bilateral, diffuse, granulomatous panuveitis triggered by an ocular penetrating injury or ophthalmic surgery in one eye. The traumatized eye is defined as the “inciting” eye, whereas the fellow eye is referred to as the “sympathizing” eye [1]. After an initial eye injury, a sight-threatening inflammation may appear in both eyes after a variable period, with symptoms occurring from 1 week to 66 years after the initial event [2]. The exact pathogenesis of SO is still not entirely known despite some research showing an autoimmune T-cell-mediated reaction against the normally sequestered ocular antigens that become exposed to the systemic immune system by ocular trauma or surgery [3].

Despite being a potentially blinding disease, the morbidity frequency measures of SO need to be better delineated in the literature as SO is challenging to study due to its rarity and often delayed presentation. Nonetheless, in a recent meta-analysis of 24 studies, He et al. found that SO’s estimated overall incidence proportion and incidence rate following open globe injury were 0.19% and 33 per 100,000 person-years, respectively [3]. Despite Marak et al. reporting the incidence of SO to be 0.1% after intraocular surgery, recent studies reported an increase in SO following surgical procedures [4].

Several surgical procedures have been reported as the primary triggers of SO, including cyclo-destructive procedures, cataract surgery, glaucoma filtration surgery, evisceration, and retinal laser photocoagulation [5]. Since the 1980s, the association between vitreoretinal (VR) surgery and SO has been investigated, and in recent years, ocular surgery, especially vitrectomy, has become an increasingly prevalent risk factor for SO [6,7,8,9,10,11,12,13,14,15,16,17,18,19,20]. However, most of these studies are heterogeneous, and they did not highlight well whether VR surgery alone or in combination with other surgical procedures in eyes with or without a history of previous ocular surgery or trauma represented the inciting event causing SO. To the best of our knowledge, no meta-analyses have investigated the morbidity frequency measures of SO triggered by single or multiple VR surgery procedures in eyes without an antecedent history of trauma and previous ocular surgery, except for previous or concomitant uneventful lens extraction. Therefore, we aimed to determine the pooled cumulative incidence of SO following single or multiple VR procedures in eyes that did not undergo either previous trauma or ocular surgery except for previous or concomitant uneventful lens extraction.

## 2. Materials and Methods

### 2.1. Search Strategy

We checked three databases from inception until 11 November 2022 (PubMed, Embase, and Scopus). The free text and controlled vocabulary were used to analyze the relationship between SO and VR surgery. Specifically, the Medical Subject Headings (MeSH) controlled vocabulary was used to search for articles in PubMed, and the Embase Subject Headings (Emtree) were used in the EMBASE. The search strategy combined the controlled vocabulary and the keywords according to the indications from each database. The keywords were selected based on readings related to the study’s subject. The controlled vocabularies and keywords were used with Boolean operators to extend and direct the search. (For addition and restriction, the Boolean operators OR and AND were used.) The investigation was conducted using recognized and extended vocabulary without database filters to achieve a significant sample with a decreased potential loss. Our core search comprised the following terms: “retinal” OR “vitreoretinal surgery” AND “sympathetic ophthalmia”. This continued until we reached a point when adding more terms provided no new results. In addition, we also hand-searched the bibliographies of included articles to identify further studies that were not found in the initial database search. The detailed search strategy and Preferred Reporting Items for Systematic Reviews and Meta-Analyses (PRISMA) Checklist are reported in Supplementary Materials S1 and S2.

### 2.2. Study Selection Data Extraction and Data Synthesis

Articles assessing the relationship between SO and VR surgery were included in this review. Specifically, we included all studies that explicitly addressed the presence of SO triggered by single or multiple VR surgery procedures in eyes without an antecedent history of trauma and previous ocular surgery, except for previous or concomitant uneventful lens extraction. 

This review is reported following the Preferred Reporting Items for Systematic Reviews and Meta-Analyses (PRISMA) guidelines [21]. Two investigators (M.R. and L.M.) independently extracted baseline and outcome data. If consensus could not be reached, the two co-authors (M.R. and L.M.) discussed the inconsistencies for adjudication. 

Articles were excluded if they were not available in the English language. In addition, all articles that did not investigate the morbidity frequency measures of SO after VR surgery procedures in eyes without an antecedent history of trauma and previous ocular surgery, except for previous or concomitant uneventful lens extraction, were excluded. Literature review studies, theses, case reports, dissertations; book chapters; technical reports; and letters from the publisher were not included in our analysis. Furthermore, studies were excluded if the study did not offer a clear description of SO assessment. Reasons for exclusion were documented. This study was registered in The International Prospective Register of Systematic Reviews (PROSPERO) (CRD42023397792). 

SO was diagnosed if there were evidence of two of the following criteria in the sympathizing eye (SE) with a history of trauma or surgery preceding the onset of uveitis: (1) bilateral anterior granulomatous or non-granulomatous uveitis (i.e., anterior segment inflammation, (2) vitritis, (3) characteristic involvement of posterior segment showing choroiditis, yellowish-white choroidal lesions (Dalen–Fuchs nodules), papillitis, vasculitis, sunset glow fundus, or exudative retinal detachment, (4) diffuse choroidal thickening in the posterior pole on B-scan ultrasonography, and (5) able to pinpoint areas of hyper- or hypo-fluorescence on fluorescein angiography with late pooling of dye.

VR surgery procedures included: scleral buckle with or without subretinal fluid (SRF) drainage, pars plana vitrectomy with or without endolaser, cryopexy, gas or silicon oil injection, pneumatic retinopexy, or a combination of them. 

We extracted the following data from each article: the first author, year published, country, study design, mean/median age of patients that developed SO after VR surgery, study period (years), total number of cases of SO in the report regardless of the main trigger, number of cases of SO after trauma in the report, number of cases of SO after VR surgery in the report (±lens extraction), VR and other surgical procedures performed, total number of VR procedures in the report, and other relevant parameters evaluated.

We used Covidence systematic review software© (Veritas Health Innovation, Melbourne, Australia), available at www.covidence.org [22], (accessed on 11 November 2022) to record and evaluate the study data between 11 October 2012 and 11 November 2022.

### 2.3. Risk of Bias Assessment 

Two authors (M.R. and L.M.) independently appraised each cross-sectional and cohort study’s methodological quality using the Newcastle–Ottawa scale (NOS) [23]. The Joanna Briggs Institute (JBI) critical appraisal checklist for the case series was used for the quality assessment of the case series [24]. Quality assessment data individually appraised by each of the reviewers were compared. M.R. and L.M. discussed the discrepancies for adjudication if consensus could not be achieved. 

### 2.4. Assessment of Quality of Evidence

The Grading of Recommendations Assessment, Development, and Evaluation (GRADE) profiler version 3.6 was used to assess the quality of evidence for each outcome, along with the consensus of two authors (M.R. and L.M.) using the GRADE system. The quality of studies is initially rated as high in this system, but it can be downgraded due to (1) bias risk, (2) inconsistency, (3) indirectness, (4) imprecision, and (5) publication bias. This system categorizes evidence into four levels of quality: high, moderate, low, and very low [25,26].

### 2.5. Statistical Analysis

A random-effects meta-analysis of pooled cumulative incidence and their 95% confidence intervals of SO triggered by single or multiple VR surgery procedures in eyes without an antecedent history of trauma and previous ocular surgery, except for previous or concomitant uneventful lens extraction among patients who developed SO regardless of the main trigger, and a random-effects meta-analysis of the pooled incidence of proportion and their 95% confidence intervals of SO triggered by single or multiple VR surgery procedures in eyes without an antecedent history of trauma and previous ocular surgery, except for previous or concomitant uneventful lens extraction among patients who underwent VR surgery were obtained based on the exact binomial distributions (i.e., number of “events” versus a number of “non-events” in a sample) with Freeman–Tukey double-arcsine transformation using the “metaprop” command in STATA (STATA Corp, College Station, TX, USA), version 17.0. To characterize potential causes of variability, we performed subgroup analyses by stratifying the data by geographic region to investigate further the proportion of surgically induced SO. 

According to Barker et al., a high I^2^ in the context of proportional meta-analysis does not necessarily mean that data are inconsistent, and the results of this test should be interpreted conservatively. Therefore, we did not perform further analysis except for subgroup analyses. Tests to evaluate publication bias, such as Egger’s test and funnel plots, were not performed as Egger’s test and funnel plots were developed in the context of comparative data, and there is no evidence that proportional data adequately adjusts for these tests [27]. Statistical significance was determined by a two-sided *p*-value of 0.05.

## 3. Results

### 3.1. Study Selection 

**Figure 1:**
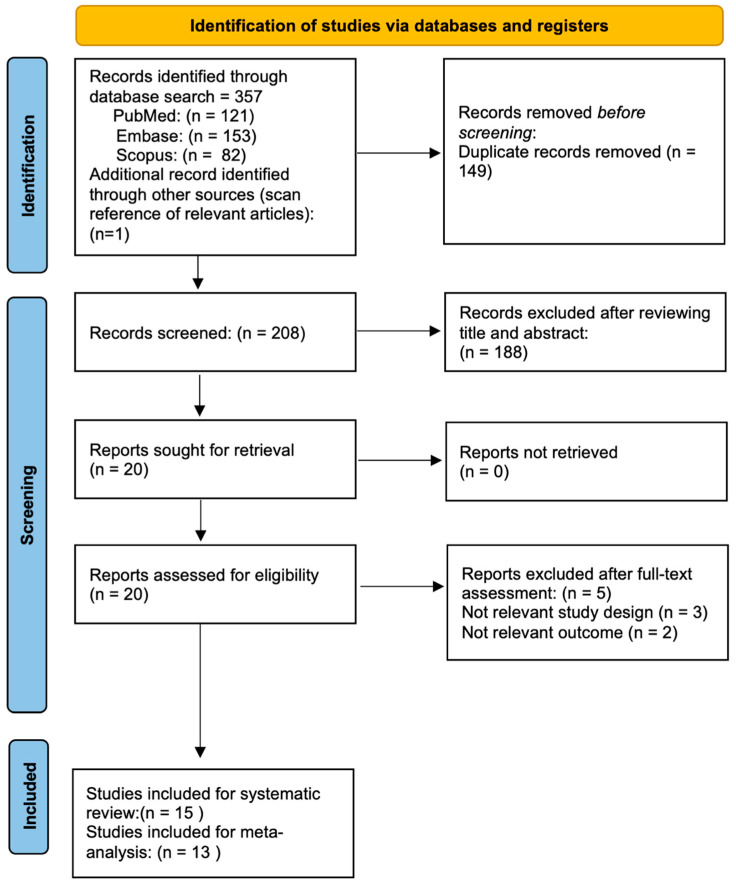
Flow diagram of the study selection process.

Figure 1 illustrates the flow chart of our analysis selection and identification process.

The search yielded 358 indexed articles (121, 153, and 82 records from PubMed, Embase, and Scopus, respectively). A search of the reference list yielded one other article. After duplication removal, we screened a total of 208 articles. After the title and abstract screening, we excluded 188 studies, and only 20 full-text studies were retrieved and assessed for final eligibility. Furthermore, an additional 5 articles were excluded because SO was neither triggered by VR surgery procedures in eyes without an antecedent history of trauma nor epidemiologic SO rates after VR surgery were investigated, resulting in 15 studies included in the systematic review [6,7,8,9,10,11,12,13,14,15,16,17,18,19,20].

Among the 15 studies included in the qualitative analysis, all [6,7,8,9,10,11,12,13,14,15,16,17,18,19,20] except two studies [11,16] reported the exact number of SO over a specific period. Indeed, these studies reported only SO’s clinical presentation, course, and outcomes following vitreoretinal surgeries. Therefore, we excluded both studies from our quantitative synthesis. Finally, the two pooled incidence of proportion meta-analyses included 13 [6,7,8,9,10,12,13,14,15,17,18,19,20] and 3 studies [9,14,20], respectively, with a total of 99 SO after VR surgery alone or with a previous or concomitant uneventful lens extraction in eyes without an antecedent history of trauma and ocular surgery.

### 3.2. Study Characteristics

A summary of the main characteristics, including the first author, year published, country, study design, mean/median age of patients that developed SO after VR surgery, study period (years), total number of cases of SO in the report regardless of the main trigger, number of cases of SO after trauma in the report, number of cases of SO after VR surgery in the report (±lens extraction), VR and other surgical procedures performed, total number of VR procedures in the report, and other relevant parameters evaluated are summarized in Table 1.

**Figure 2:**
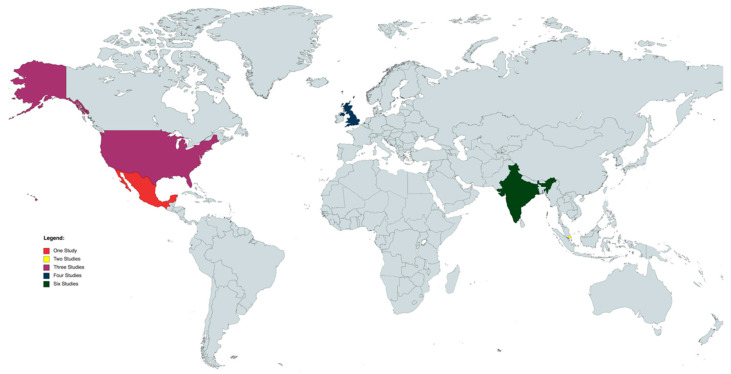
World map of studies included. Map generated through MapChart (MapChart, 2021).

We assessed eight cross-sectional studies [6,7,9,10,12,13,14,15], one longitudinal study [11], and six case series [8,16,17,18,20]. Overall, three studies recruited data exclusively from the European population [6,11,14], four exclusively from the American (north and south) populations (north and south) countries [7,9,15,16], seven exclusively from the Asian population [8,10,12,13,17,18,20], and only one study analyzed the demographic profile of patients with SO in a multicenter collaborative retrospective study whose data were retrieved from the UK, Singapore, and India [19] (Figure 2).

The review of clinical records of all patients diagnosed and treated as SO ranged from sixty [9] to two hundred and fifty-two months [12], with a total of 99 cases of SO triggered by single or multiple VR surgery procedures in eyes without an antecedent history of trauma and previous ocular surgery, except for previous or concomitant uneventful lens extraction and an overall denominator of 826 patients who developed SO regardless of the main trigger in the study period analyzed with a median age of 55.26 ± 14.74. According to the articles that reported the exact number of VR procedures (denominator), only 37 patients developed the SO after 121.511 VR operations in the study period analyzed [9,14,20].

All studies [6,7,8,9,10,11,12,13,14,15,16,17,18,19,20] except two retrieved data regardless of age. Indeed, Only Kumar et al. and Dutta Majumder et al. assessed the clinical features, management, visual outcome, and ocular complications in the sympathizing eye in pediatric patients with SO [8,13].

Only three studies reported total VR surgical operations performed over the study period, totaling 121.511 VR procedures [9,14,20].

Eleven studies reported the data of either SO after VR procedures alone or after VR surgery, plus previous or concomitant uneventful lens extraction [6,7,9,10,12,13,15,16,17,18,20]. The other four reported the total number of SO exclusively caused by VR procedures alone and uneventful lens extraction in eyes without any history of trauma and previous ocular surgery [8,11,14,19]. Five studies reported the data of SO either triggered by trauma alone or trauma followed by surgery [6,8,13,17,18].

According to data retrieved from all included studies, the total number of SO after VR procedures alone in eyes without an antecedent history of trauma and previous ocular surgery was 77, whereas the SO after VR surgery procedures in eyes that underwent VR procedures and either previous or concomitant uneventful lens extraction was 22. 

Nine studies reported the data of SO triggered by different surgical operations [6,7,9,10,12,15,16,18,19]. To be specific, only 65 cases of SO had as the main trigger different surgical procedures such as cataract surgery, glaucoma filtration surgery, diode laser trans-scleral cyclophotoablation (TCP), neodymium: yttrium–argon–garnet-(YAG)-TCP and penetrating keratoplasty (PKP) alone or combined with PPV or SB. 

Despite not being the primary purpose of the current research, trauma was the main trigger of SO in 255 cases, ranging from 0 to 94 in the included studies.

### 3.3. Meta-Analyses of Cumulative Incidence and Subgroups Meta-Analysis

A proportional random meta-analysis was performed to estimate the cumulative incidence of SO triggered by single or multiple VR surgery procedures in eyes without an antecedent history of trauma and previous ocular surgery, except for previous or concomitant uneventful lens extraction among patients who developed SO regardless of the main trigger. The total population (all patients that developed SO in the included studies) was equal to 817, and the sample size varied between 18 and 197 [6,7,8,9,10,12,13,14,15,17,18,19,20].

**Figure 3:**
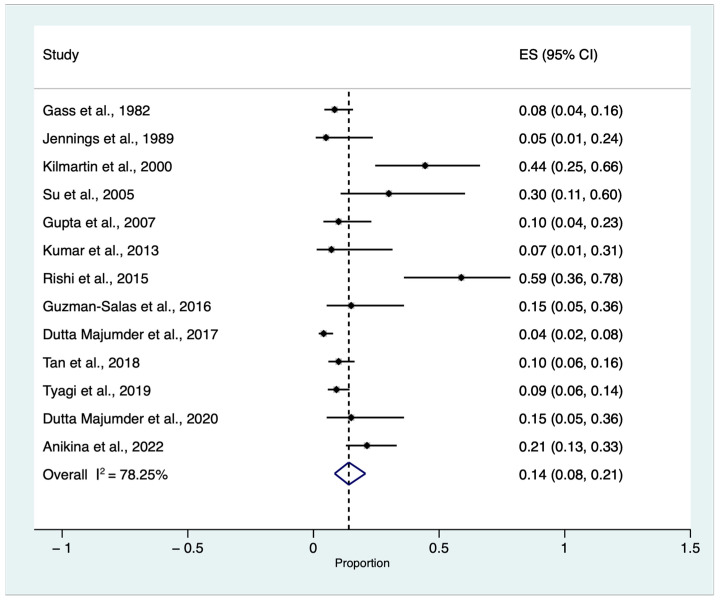
Proportional meta-analysis of cumulative sympathetic ophthalmia (SO) incidence triggered by single or multiple vitreoretinal (VR) surgery procedures in eyes without an antecedent history of trauma and previous ocular surgery, except for previous or concomitant uneventful lens extraction among patients who developed SO regardless of the main trigger [6,7,8,9,10,12,13,14,15,17,18,19,20]. ES: effect size, CI: confidence Interval.

The pooled cumulative incidence of SO triggered by single or multiple VR surgery procedures in eyes without an antecedent history of trauma and previous ocular surgery, except for previous or concomitant uneventful lens extraction among patients who developed SO regardless of the main trigger, was equal to 0.14 with a confidence interval (CI) between 0.08 and 0.21 (I^2^ = 78.25, z: 7.24, *p* < 0.01) (Figure 3).

**Figure 4:**
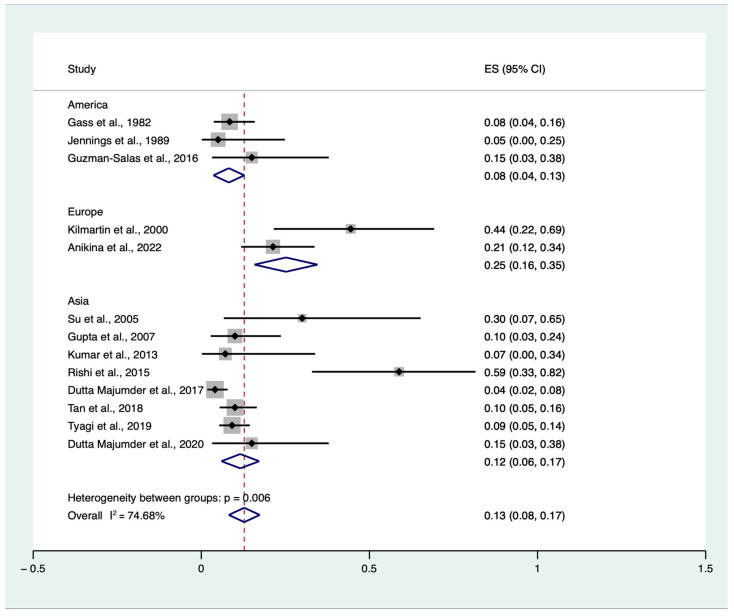
Proportional subgroup meta-analysis of cumulative sympathetic ophthalmia (SO) incidence triggered by single or multiple vitreoretinal (VR) surgery procedures in eyes without an antecedent history of trauma and previous ocular surgery, except for previous or concomitant uneventful lens extraction among patients who developed SO regardless of the main trigger according to the geographic area [6,7,8,9,10,12,13,14,15,17,18,19,20]. ES: effect size, CI: confidence interval.

Due to the expected heterogeneity among studies (I^2^ = 78.25%), we planned a subgroup analysis. Subgroup analyses stratified by categorical study-level characteristics are reported in Figure 4. Therefore, we included geographic region (Europe vs. America vs. Asia) to investigate further the cumulative incidence of SO triggered by single or multiple VR surgery procedures in eyes without an antecedent history of trauma and previous ocular surgery, except for previous or concomitant uneventful lens extraction among patients who developed SO regardless of the main trigger. One study retrieved data from three countries (India, Singapore, and the UK). As most of the data were harvested and retrieved in India (89 out of 130), we considered this study in the Asian subgroup for the analysis [19]. The test for subgroup differences indicated a statistically significant subgroup effect (*p* < 0.01), meaning that this variable statistically significantly impacts the VR-induced SO. However, despite the analysis, substantial unexplained heterogeneity existed between the subgroups (High: I^2^ = 74.68%) (Figure 4).

**Figure 5:**
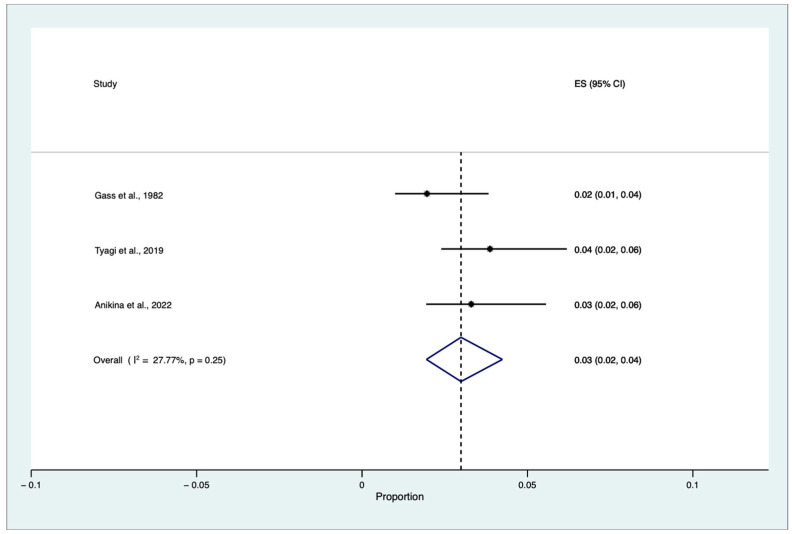
Proportional meta-analysis of cumulative sympathetic ophthalmia (SO) incidence triggered by single or multiple vitreoretinal (VR) surgery procedures in eyes without an antecedent history of trauma and previous ocular surgery, except for previous or concomitant uneventful lens extraction among patients who underwent VR surgery [9,14,20]. ES: effect size, CI: confidence interval.

A proportional random meta-analysis was performed to estimate the pooled cumulative incidence of SO triggered by single or multiple VR surgery procedures in eyes without an antecedent history of trauma and previous ocular surgery, except for previous or concomitant uneventful lens extraction among patients who underwent VR procedures. The total population was equal to 121.511, and the sample size varied between 39,391 and 41,365. The pooled cumulative incidence of SO triggered by single or multiple VR surgery procedures in eyes without an antecedent history of trauma and previous ocular surgery, except for previous or concomitant uneventful lens extraction among patients who underwent VR surgery, was equal to 0.03 for every 100 people, with a confidence interval (CI) between 0.02% and 0.004% (I^2^ = 27.77, z: 9.11, *p* = 0.25) (Figure 5).

### 3.4. Risk of Bias and GRADE Assessment

Tables S1 and S2, available in Supplementary Material S3, summarize all studies’ risk of bias evaluation. Most studies scored 1 or 2 in the major domains of the quality scale used. The quality rating of the cross-sectional studies averaged 7.5 (95% CI 7.13 to 7.86) of the maximum score on the Newcastle–Ottawa Scale [6,7,9,10,12,13,14,15]. The quality rating of the longitudinal study [11] averaged 7 (95% CI 7 to 7) of the maximum score on the Newcastle–Ottawa Scale. 

Overall, four cross-sectional studies reached a total score between 8 and 10 [6,9,10,14] and four reached a total score of 7 [7,12,13,15]. The longitudinal research achieved a score of 7 out of 9. According to the JBI critical appraisal checklist for case series, the quality of the included studies was moderate to good. All case series scored 7 out of 10 quality criteria or higher. Notably, two case series scored 8 out of 10 quality criteria as they extensively provided information regarding population demographics [19,20].

The quality of evidence for our primary outcome (pooled cumulative incidence of SO triggered by single or multiple VR surgery procedures in eyes without an antecedent history of trauma and previous ocular surgery, except for previous or concomitant uneventful lens extraction among patients who developed SO regardless of the main trigger) was low according to the GRADE methodology (Table S3 available in Supplementary Material S3.

**Table 1:** Characteristics of studies included in the systematic review.

Study Name	Year of Publication	Country	Age of SO Patients after VR Surgery (Average ± SD)	Study Design	Duration (y.)	Number of SO Cases in the Study	Number of Cases of SO after Trauma (N and %) in the Study	Number of Cases of SO after Surgery (N and %) in the Study	Number of Cases of SO after VR Surgery (±Lens Extraction) (N and %) in the Study	Number of Cases of SO after Other Surgeries (N and %) in the Study	VR Procedures	Other Surgeries:	Total Number of VR Procedures in the Study	Other Relevant Parameters Evaluated:
Gass et al. [9]	1982	USA	52 ± 13.98	Cross-Sectional	Five years	1. Survey of eye pathology laboratories: 53 2. Armed Forces Institute of Pathology: 33 3. Survey of retinal surgeons: 9	1. Survey of eye pathology laboratories: 29 (54.7%) 2. Armed Forces Institute of Pathology: not stated 3. Survey of retinal surgeons: 3 (33.3%)	1. Survey of eye pathology laboratories: 24 (45.3%) 2. Armed Forces Institute of Pathology: 1 (3%) 3. Survey of retinal surgeons: 4 (44.4%)	1. Survey of eye pathology laboratories: Exclusively Retinal Surgery: 3 (5.7%) Retinal Surgery + Lens extraction: 3 (5.7%) 2. Armed Forces Institute of Pathology: Retinal Surgery + Lens extraction: 1 (3%) 3. Survey of retinal surgeons: Exclusively Retinal Surgery: 1 (11.1%)	1. Survey of eye pathology laboratories: 14 (26.4%) 2. Armed Forces Institute of Pathology: not stated 3. Survey of retinal surgeons: not stated	1. Survey of eye pathology laboratories: SB: 3 (5.7%) 2. Armed Forces Institute of Pathology: 0 3. Survey of retinal surgeons: PPV: 1 (11.1%)	1. Survey of eye pathology laboratories: Cataract extraction. 10 Filtering operation 3, Combined cataract extraction and filtering operation 1 2. Armed Forces Institute of Pathology: not stated 3. Survey of retinal surgeons: not stated	1. Survey of eye pathology laboratories: 22.840 (surgical specimens) 2. Armed Forces Institute of Pathology: 3.000 eyes 3. Survey of retinal surgeons: 14.915 vitrectomies	/
Jennings et al. [7]	1989	USA	62.3 ± 7.76	Cross-Sectional	11-year period: 1974 to 1985	20	16 (80%)	3 (15%)	Exclusively Retinal Surgery: 1 (5%)	2 (10%)	PPV: 1 (5%)	2 (10%) Extracapsular Extraction with vitreous loss, 2 steroid injections, and 2 vitrectomies. Extracapsular Extraction with a dropped nucleus.	Not stated	SO from onset to last Observation in patients after VR surgery: 12.33 ± 14.46 months
Kilmartin et al. [6]	2000	UK	66 ± 10.2	Cross-Sectional	July 1997 to September 1998, 14 months	18	Exclusively Trauma: 6 (33%) Trauma + Surgery: 2 (11.1%)	10 (56%)	Exclusively Retinal Surgery: 6 (33%) 3 of these patients having undergone just one PPV Retinal Surgery + Lens extraction: 2 (11.1%)	3 (16.7%)	PPV RD: 5 (27.8%) Ext RD: 1 (5.5%)	Trabeculectomy: 1 (5.5%) Ext beam DTX, PPV X2, Enucl: 1 (5.5%) Ext RD, PPV RD, Cyclodiodetx: 1 (5.5%)	Not stated	/
Pollack et al. [16]	2001	USA	48.87 ± 21.79	Case series	Not stated	8	0	8 (100%)	Exclusively Retinal Surgery: 1 (12.5%) Retinal Surgery + Lens extraction: 6	1 (12.5%)	PPV: 1 PPV + SB + Lens Extraction:6	Tectonic PKP, PPV: 1 (12.5%)	Not Stated	Time from PPV to onset of symptoms of SO: median of 7 months. Follow-up from onset of symptoms: 10.5 months Initial VA in sympathizing eye: 0.67 ± 0.56 LogMAR Final VA in the sympathizing eye: 0.47 ± 0.59
Grigoropoulos et al. [11]	2006	UK	Not stated	Cohort	Not stated	1	0	0	1	0	PPV + 210° retinectomy.	None	1142 operations performed on the 304 eyes.	VA was limited to PL and the eye was hypotonus Thirty-one months after the initial procedure and 9 months after the last procedure, the fellow eye developed SO VA in the fellow eye decreased from 6/9 to 6/18 and remained stable
Su et al. [18]	2005	Singapore	63 ± 19.98	Case series	1993–2003, ten years	10 (1.08%)	Exclusively Trauma: 1 (10%) Trauma + Surgery: 3 (30%)	6 (60%)	Exclusively Retinal Surgery: 1 (10%) Retinal Surgery + Lens extraction: 2 (20%)	3 (30%)	Not stated	TCP: 2 YAG-TCP: 1	924	Retinal surgery patients: Patient 1: initial VA in SE: 0.4 LogMAR Final VA in SE: 0.7 LogMAR Patient 2: initial VA in SE: 0.4 LogMAR Final VA in SE: 0.5 LogMAR Patient 3: initial VA in SE: HM Final VA in SE: NPL Interval between IE and onset of symptoms: (mean ± SD): 29 ± 32.51 months
Gupta et al. [10]	2007	India	Not stated	Cross-Sectional	June 1989–August 2004, 15 years and 2 months	40	30 (75%)	10 (25%)	Exclusively Retinal Surgery: 4 (10%)	6 (15%)	PPV: 2 (5%) SB: 2 (5%)	Lens extraction: 5 (12.5%) Glaucoma Filtration Surgery: 1 (2.5%)	Not stated	/
Kumar et al. [13]	2013	India	Not stated (pediatric age)	Cross-Sectional	2001–2011, ten years	14	Exclusively Trauma:13 (92.9%)	1 (7.1%)	Exclusively Retinal Surgery: 1 (7.1%)	0	PPV: 1 (7.1%)	/	2511 pediatric patients with open globe injuries	/
Rishi et al. [17]	2015	India	39.4 ± 14.72	Comparative case series	1995–2011, 16 years	17	Trauma + VR surgery: 7 (41.2%)	10 (58.8%)	Exclusively Retinal Surgery: 5 (29.4%) Retinal Surgery + Lens extraction: 5 (29.4%)	0	SB: 3 SB + PPV: 2	None	Not stated	Initial VA in SE (mean ± SD): 0.78 ± 0.72 Final VA in SE (mean ± SD): 0.26 ± 0.55 Follow-up (mean ± SD): 45 ± 52.74 months Duration of symptoms 22.5 days Interval between surgery and SO (mean ± SD): 38.1 ± 52.79 months Average Follow-up period 34 months
Guzman-Salas et al. [15]	2016	Mexico	Not Stated	Cross-Sectional	2007–2013, 6 years	20	10 (50%)	10 (50%)	Exclusively Retinal Surgery: 3 (15%)	7 (45%)	Retinopexy	Lens Extraction: 6 (30%) Ahmed valve implantation 1 (5%)	Not Stated	/
Dutta Majumder et al. [12]	2017	India	Not Stated	Cross-Sectional	June 1994–November 2015: 21 years and 5 months	197	Not Stated	14 (7.1%)	Exclusively Retinal Surgery: 8 (4.1%)	6 (3%)	SB: 4 (2%) PPV: 4 (2%)	Lens Extraction: 1 (0.5%) Lens Extraction + Anterior Vitrectomy: 3 (1.5%) Trabeculectomy: 1 (0.5%) PKP: 1 (0.5%)	Not Stated	/
Tyagi et al. [20]	2019	India	41.14 ± 16.53	Retrospective case series	2005–2015, ten years	175	0	16 (9.1%)	Exclusively Retinal Surgery: 13 (7.4%) Retinal Surgery + Lens extraction: 3 (1.7%)	Not Stated	PPV: 8 (4.6%) SB + PPV: 5 (2.9%)	Not Stated	41.365 PPV	Time interval from surgery to diagnosis: (mean): 154 days 2. VA: Initial VA in SE (mean ± SD): 1.03 ± 0.56 LogMar Final VA in SE (mean ± SD): 0.43 ± 0.57 LogMar Duration of follow-up of (mean) 25.8 months Duration from surgery (days): 194.5 ± 349.28
Tan et al. [19]	2018	India UK Singapore	Not Stated	Retrospective Multicenter Case Series	1995–2014, 9 years	130	94 (72.3%)	36 (27.9%)	13 (36.1%)	23	Not Stated	Lens Extraction 11 (30.5%) Glaucoma surgery 6 (16.7%) Others: 6 (16.7%)	Not Stated	/
Dutta Majumder et al. [8]	2020	India	Not Stated (Pediatric Age)	Retrospective Case Series	December 1997–January 2017, 19 years, 1 month	20	Exclusively Trauma: 13 (65%) Trauma + Surgery: 4 (20%)	3 (15%)	3 (15%)	0	Not Stated	None	Not Stated	/
Anikina et al. [14]	2022	UK		Cross-Sectional	January 2000 and December 2015, 15 year period	61	40 (65.6%)	21 (34.4%) as main trigger	13 (21.3%) as main trigger	Not Stated	10 multiple procedures: SB: 6 PPV: 21 (1 of the cases involved a combination).	Not Stated	39.391 VR procedures SO after a single VR procedure was estimated to be 0.008%, rising to 6.67% with 7 procedures	/

Abbreviations: DTX: radiotherapy, PPV: pars plana vitrectomy, Enucl = enucleation, Cyclodiodetx = cyclodiodetherapy, RD: retinal detachment, Ext = external, PKP: penetrating keratoplasty, SB: scleral buckle, LogMAR: Logarithm of the Minimum Angle of Resolution, PL: perception light, TCP: diode laser trans-scleral cyclophotoablation, Yag: neodymium:yttrium–argon–garnet, HM: hand movement, NPL: non-perception light, VR: vitreoretinal, SO: sympathetic ophthalmitis, SD: standard deviation, SE: sympathizing eye, IE: inciting event, y: years, N: number; VA: visual acuity.

## 4. Discussion

Our systematic review and meta-analysis aimed to evaluate the morbidity frequency measures in terms of the cumulative incidence of SO triggered by single or multiple VR surgery procedures in eyes without an antecedent history of trauma and previous ocular surgery, except for previous or concomitant uneventful lens extraction, and to further investigate the relationship between VR surgery and SO. 

Analyzing data from thirteen studies, we found that the cumulative incidence of SO after single or multiple VR surgery in eyes with no history of trauma or previous ocular surgery, except for previous or concomitant uneventful lens extraction, was equal to 14% (CIs: 0.08–0.21%, *p* < 0.01) among patients who developed SO regardless of the main trigger [6,7,8,9,10,12,13,14,15,17,18,19,20].

The proportion of surgically induced SO has been increasing over the years, as previously stated by Su et al. [18], who found that SO occurred in 70% of their patients following ocular surgery, especially VR. Indeed, uveal protein release might occur during different VR surgical steps, such as the creation of sclerotomies, cryo-retinopexy, or subretinal and fluid drainage. This trend has increased, according to data reported by Hakin et al. [28], Jennings et al. [7], and Kilmartin et al. [6], from 17% to 56% from 1974 to 1998. Tan et al. also observed this increasing trend [19]. Among several reasons, the increase in surgically induced SO could be attributed to the advancements in vitreoretinal surgery. Indeed, more cases that would have previously been dismissed are now being operated on by ophthalmologists, including complicated cases that require multiple surgeries or procedures. The high level of advancement in VR surgery has also raised patient expectations, and more patients are likely to undergo numerous procedures on the same eye. In contrast, in their retrospective analysis, Dutta et al. [8], evaluating the clinical pattern of postsurgical SO in a tertiary eye care center in India, found a relatively lower proportion of surgically induced SO (7.10% had surgically induced SO). These data were consistent with those of Kumar et al. [13], who found a proportion of surgically induced SO of 7.1% in the pediatric population in an Indian center. This may be partially explained, considering that most Indian people reside in rural areas, where they often have poor access to personal protective equipment at work and have disproportionately low levels of awareness of eye safety. According to these data, our subgroup meta-analysis showed a higher rate of VR-induced SO in European countries compared to Asian countries, 25% (CIs: 0.16–0.35) vs. 12% (CIs: 0.06–0.17), *p* < 0.01. No reliable data could be obtained from American countries, as most of the studies were conducted before 1990 [7,9], and only in that year, de Juan and Hickingbotham developed the first 25-gauge (0.5 mm diameter) vitrectomy system based on conventional sclerotomy methods [29].

Unfortunately, repeated surgical procedures result in higher levels of uveal protein release. In 2022, Anikina et al. supported the increased proportion of surgically induced SO, analyzing the role of multiple VR surgery procedures and the type of VR intervention. They found that only eight (13%) patients had undergone a single event before their SO diagnosis. Specifically, VR surgery was performed before the diagnosis of SO in 25 of 61 cases, representing 41% of their entire cohort [14]. In addition, they suggested that the greater-than-average surgical complexity in VR surgery was responsible for many cases of VR-induced SO. Indeed, out of 25 VR procedures performed, there was a 29% retinectomy rate of PPV and a 57% rate of the use of silicone oil tamponade, and the higher number of procedural steps as well as the increased length of surgical time allowed a higher ocular antigen exposure to the immune system, potentially leading to SO. 

With regard to different numbers of VR procedures, the authors were the first to analyze the effect of multiple VR procedures on the incidence of SO, demonstrating that performing two VR procedures on a patient raised the incidence of SO by a ten-fold increase in comparison to only performing one VR procedure on a patient. There was an exponential increase in risk according to the number of procedures, with 6.67% of patients who underwent seven VR procedures developing SO. Furthermore, despite pars plana vitrectomy being associated with twice the risk as compared to external retinal detachment repair surgeries [30], Dutta et al. [12] found that the incidence of SO following pars plana vitrectomy and scleral buckle surgery was the same, as previously published by Gupta et al. [10]. Indeed, patients undergoing scleral buckle surgery also face the risk of developing SO, as these procedures are usually associated with subretinal drainage, which may expose uveal antigens [12].

The pooled cumulative incidence of SO triggered by single or multiple VR surgery procedures in eyes without an antecedent history of trauma and previous ocular surgery, except for previous or concomitant uneventful lens extraction among patients who underwent VR surgery procedures, was equal to 0.03% (CIs: 0.004–0.02%, *p* = 0.25). Our results are consistent with the most recent data retrieved and published by Anikina et al., who analyzed 39,391 VR procedures performed over 15 years. They found 13 cases of SO triggered by VR surgery alone, corresponding to 1 in 3030 (0.03%) cases [14]. Accordingly, in 2019 Tyagi et al. found 16 cases of SO after VR surgery alone, corresponding to 1 in 2585 (0.04%) cases [20].

One of our systematic review and meta-analyses’ strengths is that we included many studies retrieving data from patients of different countries covering a considerable period, making our findings generalizable. Moreover, we systematically evaluated all reports without timespan restriction, analyzing data from 40-year time studies. Our subgroup analyses did not significantly alter our results. Nevertheless, this systematic review and meta-analysis has several limitations. First, we limited our literature search to the English language, and no articles in Chinese and Japanese, including data from those populations, were retrieved. Therefore, the Asian subgroup mostly included articles whose data were retrieved in South Asia. Second, we only included thirteen studies in our meta-analysis. Third, we included in our meta-analysis, data of patients regardless of age combining data from the pediatric and adult populations; fourth, the meta-analysis included data collected after hand-searching the numerical data that could increase the risk of biases. Fifth, we found high heterogeneity, which our subgroup meta-analysis model only partially explained. This implies that other unknown sources of heterogeneity were present and may have heavily biased our results. Sixth, we recruited studies from different periods where the surgical instruments and techniques differed according to the technological advances of that era. Seventh, most of the included studies were retrospective and had intrinsic limitations, producing biased or inaccurate estimates. Eighth, due to the nature of the included studies, heterogeneous incidence rates, selection bias from different types of VR surgeries and VR diseases, and the inclusion of survey studies that are not methodologically comparable to retrospective studies could be an additional source of biases. In addition, in the oldest studies, the definition of SO was elusive or ill-defined, particularly in cases that manifested long after the surgery. The follow-up times in each study were variable, although SO can develop up to 66 years after the inciting injury. Finally, we synthesized data from patients that underwent single or multiple VR surgeries.

## 5. Conclusions

To the best of our knowledge, this is the first meta-analysis synthesizing the morbidity frequency measures of SO triggered by single or multiple VR surgery procedures in eyes without an antecedent history of trauma and previous ocular surgery, except for previous or concomitant uneventful lens extraction.

Although postsurgical sympathetic ophthalmitis is rare, it is a bilateral blinding disease. Despite its success and low incidence rate, VR surgery should be viewed as a possible inciting event for SO. Hence, ophthalmologists may consider counseling patients who require surgical single or multiple procedures about this risk. This could lead to better decision making and a more accurate process of consent for surgery.

## Data Availability

Not applicable.

## Supplementary Materials

The following supporting information can be downloaded at: https://www.mdpi.com/article/10.3390/jcm12062316/s1, Supplementary Material S1: Detailed Research Strategy Material; Supplementary Material S2: PRISMA Checklist [31]; Supplementary Material S3: Risk of bias evaluation and GRADE assessment.
