# Supplementary material for: Sympathetic Ophthalmia after Vitreoretinal Surgery without Antecedent History of Trauma: A Systematic Review and Meta-Analysis
Source: J Clin Med. 2023 Mar 16;12(6):2316. doi: 10.3390/jcm12062316 (PMC10057773; doi:10.3390/jcm12062316)
Supplement: Supplementary file 1 [file jcm-12-02316-s001.zip › Supplementary Material S1.pdf]

Supplementary Material S1: Detailed Search Strategy

PUBMED:
Free Text
Initial search performed on 11 October 2022
Search updated on 11 November 2022
Language: English

#	**Search Term**	**No. Results**
**1**	"retinal" OR "vitreoretinal"	264.704
**2**	"surgery”	4.546.726
**3**	1 AND 2	46.362
**4**	" sympathetic"	92.237
**5**	“Ophthalmia”	11.287
**6**	4 AND 5	707
**7**	3 AND 6	121

Controlled Vocabulary: Medical Subject Headings (MeSH)
Initial search performed on 11 October 2022
Search updated on 11 November 2022
Language: English

#	**Search Term**	**No. of Results**
**1**	("Vitreoretinal Surgery"[MeSH]) AND "Sympathetic Ophthalmia"[MeSH]	6

All MeSH search results had already been found in the free-text search.

EMBASE

Free Text
Initial search performed on 11 October 2022
Search updated on 11 November 2022
Language: English

Mapping options enabled:

- map to preferred term in Emtree
- search also as free text in all fields
- explode using narrower Emtree terms
- search as broadly as possible

#	**Search Term**	**No. of Results**
**1**	(((((retinal) OR (vitreoretinal)) AND (surgery)) AND (sympathetic)) AND (ophthalmia))	153

SCOPUS

Free Text
Initial search performed on 11 October 2022
Search updated on 11 November 2022
Language: English

#	Search Term	**No. of Results**
**1**	(TITLE-ABS-KEY((“retinal" OR "vitreoretinal))) AND (TITLE-ABS-KEY "surgery"))) AND (TITLE-ABS-KEY (("sympathetic ") AND (TITLE-ABS-KEY(("ophthalmia ")	82
