# Supplementary material for: Sympathetic Ophthalmia after Vitreoretinal Surgery without Antecedent History of Trauma: A Systematic Review and Meta-Analysis
Source: J Clin Med. 2023 Mar 16;12(6):2316. doi: 10.3390/jcm12062316 (PMC10057773; doi:10.3390/jcm12062316)
Supplement: Supplementary file 1 [file jcm-12-02316-s001.zip › Supplementary Material S3.pdf]

Table S1: Newcastle–Ottawa Scale for Critical Appraisal of Cross-Sectional and Longitudinal Studies

		Selection				Comparability	Outcome		
**Source**	**Study design**	**Representativeness of the sample**	**Sample size**	**Non-respondents**	**Ascertainment of exposure**	**Based on design and analysis**	**Assessment of outcome**	**Statistical test**	**Total score**
Gass et al., 1982 [9]	Cross-sectional	+1 (b)	+1 (a)	+1 (a)	+1 (b)	+1 (a)	+1 (b)	+1 (a)	8/10
Jennings et al., 1989 [7]	Cross-sectional	+1 (b)	+0 (b)	+1 (a)	+2 (a)	+1 (a)	+1 (b)	+1 (a)	7/10
Kilmartin et al., 2000 [6]	Cross-sectional	+1 (b)	+1 (a)	+1 (a)	+2 (a)	+1 (a)	+1 (b)	+1 (a)	8/10
Gupta et al., 2007 [10]	Cross-sectional	+1 (b)	+1 (a)	+1 (a)	+2 (a)	+1 (b)	+1 (b)	+1 (a)	8/10
Kumar et al., 2013 [13]	Cross-sectional	+1 (b)	+1 (a)	+1 (a)	+2 (a)	+1 (a)	+1 (b)	+1 (a)	7/10
Guzman-Salas et al., 2016 [15]	Cross-sectional	+1 (b)	+0 (b)	+1 (a)	+2 (a)	+1 (a)	+1 (b)	+1 (a)	7/10
Dutta Majumder et al., 2017 [12]	Cross-sectional	+1 (b)	+1 (a)	+1 (a)	+1 (b)	+1 (a)	+1 (b)	+1 (a)	7/10
Anakina et al., 2022 [14]	Cross-sectional	+1 (a)	+1 (a)	+1 (a)	+2 (a)	+1 (a)	+1 (b)	+1 (a)	8/10

		Selection				Comparability	Outcome			
**Source**	**Study design**	**Representativeness of the exposed cohort**	**Selection of the non-exposed cohort**	**Ascertainment of exposure**	**Outcome was not present at start**	**Based on design and analysis**	**Assessment of outcome**	**Follow up long enough**	**Adequacy of follow up**	**Total score**
Grigoropoulos et al., 2006 [11]	Longitudinal	+1 (b)	+1 (a)	+1 (a)	+1 (a)	+0 (c)	+1 (b)	+1 (a)	+1 (b)	7/9

Representativeness of the sample: a) Truly representative of the average in the target population. b) Somewhat representative of the average in the target population; c) Selected group of users) No description of the sampling strategy; Sample size: a) Justified and satisfactory. b) Not justified; Non-respondents: a) Comparability between respondents and non-respondents characteristics is established, and the response rate is satisfactory. b) The response rate is unsatisfactory, or the comparability between respondents and non-respondents is unsatisfactory. c) No description of the response rate or the characteristics of the responders and the non-responders; Ascertainment of the exposure: a) Validated measurement tool. b) Non-validated measurement tool, but the tool is available or described. c) No description of the measurement tool; Comparability: 1) The subjects in different outcome groups are comparable, based on the study design or analysis. Confounding factors are controlled. a) The study controls for the most important factor (select one). b) The study control for any additional factor; Outcome: 1) Assessment of the outcome: a) Independent blind assessment; b) Record linkage; c) Self report; d) No description. 2) Statistical test: a) The statistical test used to analyze the data is clearly described and appropriate, and the measurement of the association is presented, including confidence intervals and the probability level (p value); b) The statistical test is not appropriate, not described or incomplete.

Table S2: Quality assessment of the included case series using the Joanna Briggs Institute (JBI) Critical Appraisal Checklist for Case Series

Source	Were there clear criteria for inclusion in the case series?	Was the condition measured in a standard, reliable way for all participants included in the case series?	Were valid methods used for identification of the condition for all participants included in the case series?	Did the case series have consecutive inclusion of participants?	Did the case series have complete inclusion of participants?	Was there clear reporting of the demographics of the participants in the study?	Was there clear reporting of clinical information of the participants?	Were the outcomes or follow up results of cases clearly reported?	Was there clear reporting of the presenting site(s)/clinic(s) demographic information?	Was statistical analysis appropriate?
Pollack et al., 2001 [16]	yes	yes	yes	no	yes	yes	yes	yes	no	not applicable
Su et al., 2005 [18]	yes	yes	yes	no	yes	yes	yes	yes	no	not applicable
Rishi et al. 2015 [17]	yes	yes	yes	no	yes	yes	yes	yes	no	appropriate
Tyagi et al., 2019 [20]	yes	yes	yes	no	yes	yes	yes	yes	yes	not applicable
Tan et al., 2018 [19]	yes	yes	yes	no	yes	yes	yes	yes	yes	appropriate
Dutta Majumder et al., 2020 [8]	yes	yes	yes	no	yes	yes	yes	yes	no	not applicable

Table S3: GRADE assessment

Certainty assessment								Patients (n)		Effect	Certainty
Outcome	Studies (n)	Study design	Risk of bias	Inconsistency[a]	Indirectness[d]	Imprecision[c]	Publication bias[b]	Number of Cases of SO after VR Surgery (± Lens Extraction)	Total	Relative MD or OR (95% CI) or PCI	
Cumulative incidence of SO triggered by single or multiple VR surgery procedures in eyes without an antecedent history of trauma and previous ocular surgery, except for previous or concomitant uneventful lens extraction among patients who developed SO regardless of the main trigger [6–10,12–15,17–20].	13	observational studies	not serious	serious	not serious	Not serious	Undetected	91	817*	**0.14** (0.08 to 0.21)	⊕⊕◯◯ Low
Cumulative incidence of SO triggered by single or multiple VR surgery procedures in eyes without an antecedent history of trauma and previous ocular surgery, except for previous or concomitant uneventful lens extraction among patients who underwent VR procedures [9,14,20].	3	observational studies	not serious	serious	not serious	Not serious	Undetected	37	121511**	**0.03%** (0.02% to 0.04%)	⊕⊕◯◯ Low

CI: Confidence interval; **MD:** Mean difference; **PCI:** pooled cumulative incidence, **OR:** Odds ratio; SO: sympathetic ophthalmitis; VR: vitreoretinal; [a] Substantial heterogeneity I^2 > 60% (serious) or >90% (very serious); [b] Strongly suspected if funnel plot suggestive of publication bias or lack of small studies and negative effects; [c] serious if a total number of events is less than 300, CIs overlap or non clinically significant effect; [d] Serious indirectness refer to variation of outcome measure or definition across studies, *Total number of Cases of SO, ** Total Number of VR Procedures.
